# A phase II study of chemotherapy in combination with telomerase peptide vaccine (GV1001) as second-line treatment in patients with metastatic colorectal cancer

**DOI:** 10.7150/jca.70385

**Published:** 2022-02-14

**Authors:** Sejin Kim, Bum Jun Kim, Ilhwan Kim, Jung Han Kim, Hee Kyung Kim, Hyewon Ryu, Dae Ro Choi, In Gyu Hwang, Hunho Song, Jung Hye Kwon, Joo Young Jung, Boram Han, Dae Young Zang

**Affiliations:** 1 Division of Hematology-Oncology, Department of Internal Medicine, Hallym University Medical Center, Hallym University College of Medicine, Anyang-si, Republic of Korea.; 2 Division of Oncology, Department of Internal Medicine, Haeundae Paik Hospital, Inje University College of Medicine, Busan, Republic of Korea.; 3 Department of Internal Medicine, Chungbuk National University College of Medicine, Cheongju, Republic of Korea.; 4 Division of Hematology and Oncology, Department of Internal Medicine, Chungnam National University Hospital, Chungnam National University College of Medicine, Daejeon, Republic of Korea.; 5 Department of Internal Medicine, Chung-Ang University Hospital, Chung-Ang University College of Medicine, Seoul, Republic of Korea.

**Keywords:** Colorectal neoplasm, cancer vaccines, GV1001

## Abstract

**Background:** GV1001 is a human telomerase peptide vaccine that induces a CD4/CD8 T-cell response against cancer cells, thereby affording an immunological anti-tumor effect. Here, we evaluated the efficacy and safety of GV1001 in combination with chemotherapy in patients with metastatic colorectal cancer who had failed first-line chemotherapy.

**Methods:** This multicenter, non-randomized, single-arm phase II study recruited recurrent or metastatic colorectal cancer patients with measurable disease who had failed first-line chemotherapy. Patients received GV1001 and chemotherapy concomitantly based on a pre-established schedule. Cytotoxic chemotherapy and targeted agents (bevacizumab, cetuximab, or aflibercept) were allowed to be used at the discretion of the investigator. The primary endpoint was the disease control rate; secondary endpoints were the objective response rate, progression-free survival, overall survival, and safety outcomes. The baseline serum eotaxin level (a potential predictive biomarker of GV1001) was analyzed. To determine whether an adequate immune response had been induced, a delayed-type hypersensitivity test and a T-cell proliferation test were performed.

**Results:** From May 13, 2015 to October 13, 2020, 56 patients with recurrent or metastatic colorectal cancer treated in seven hospitals of South Korea were enrolled. The median patient age was 64 years (range, 29-82 years); 67.9% were men. Of all patients, 66.1% had left-side colorectal cancer and the RAS mutation was present in 25%. The disease control rate and the objective response rates were 90.9% (95% confidence interval [CI]: 82.4-99.4%) and 34.1% (95% CI, 20.1-48.1%), respectively. The median progression-free survival was 7.1 months (95% CI, 5.2-9.1 months) and the median overall survival was 12.8 months (95% CI, 9.9-15.8 months). The most common all-grade adverse events were neutropenia (48.2%), nausea (26.8%), neuropathy (25.0%), stomatitis (21.4%), and diarrhea (21.4%). Immune response analysis showed that no patient had positive delayed-type hypersensitivity test results; antigen-specific T-cell proliferation was observed in only 28% of patients. The baseline eotaxin level was not associated with any efficacy outcome.

**Conclusion:** Although no clear GV1001-specific immune response was observed, the addition of GV1001 vaccination to chemotherapy was tolerable and associated with modest efficacy outcomes.

## Introduction

Colorectal cancer (CRC) is the third most common cancer and the second leading cause of cancer death worldwide 1. Although the overall mortality of CRC continues to decline if the disease is operable, the survival outcomes of metastatic disease remain dismal. Chemotherapy in combination with targeted monoclonal antibodies has become the main treatment modality for inoperable disease; however, immunotherapy has recently changed the treatment paradigm for metastatic CRCs. After pembrolizumab, an anti-programmed death 1 (PD-1) immune checkpoint inhibitor, exhibited a significant clinical benefit in patients with mismatch repair-deficient (dMMR) CRCs 2, many immune checkpoint inhibitors were investigated. At the 2021 American Society of Clinical Oncology (ASCO) annual meeting, pembrolizumab first-line therapy was reported to be superior to chemotherapy in patients with dMMR CRCs 3. Anti-cancer vaccination is another type of immunotherapy; many vaccines (including peptide, autologous, and dendritic cell vaccines) have been tested in CRC patients. However, no such vaccine has exhibited a clinical benefit thus far 4-6.

Telomerase (a telomere-repair enzyme) is expressed in 85-90% of human solid cancers 7; thus, it is an attractive target for anti-cancer treatment. In normal cells, the telomeric ends of DNA become progressively shortened with repeated cell division; the cells eventually enter replicative senescence 8. However, cancer cells avoid such senescence, becoming immortal through the reactivation of telomerase. This has a crucial role in the oncogenic transformation of many cancers, including CRCs 9, 10.

GV1001 is a human telomerase, 16-amino acid peptide vaccine derived from the reverse transcriptase subunit. GV1001 induces a CD4/CD8 T-cell response against cancer cells, yielding an immunological anti-tumor effect 11. After a GV1001-specific immune response and promising efficacy results were obtained in early-stage clinical studies of patients with pancreatic and non-small cell lung cancers 12, 13, a large-scale study evaluating the synergistic effects of GV1001 and conventional chemotherapy in pancreatic cancer patients was conducted, but failed to prove the benefit over chemotherapy alone 14. However, in the subgroup analysis, patients with high baseline eotaxin level were significantly associated with better overall survival (OS) with GV1001 vaccination 15. At the 2021 ASCO annual meeting, synergistic effects of GV1001 and conventional chemotherapy were reported in pancreatic cancer patients with high eotaxin levels 16. The median OS significantly improved after GV1001 vaccination plus chemotherapy compared to chemotherapy alone (11.3 months [95% CI, 8.6-14.0 months] vs. 7.5 months [95% CI, 5.1-10.0 months], p = 0.021).

GV1001 has also been investigated in advanced melanoma 17, B-cell chronic lymphocytic leukemia 18, cutaneous T-cell lymphoma 19 and has shown modest efficacy outcome with induction of immune response. However, to date, GV1001 has not been investigated in patients with advanced CRCs.

Most (approximately 85% of patients) CRCs have chromosomal instability (CIN), while other CRCs have a high grade microsatellite instability (MSI) phenotype, and telomere dysfunction may be considered a major driving mechanism of CIN development 10. Consistent results that increased telomerase activity is associated with tumor progression and poor survival have been reported 20, and these results provides a theoretical background for investigating GV1001, a telomerase peptide vaccine, in patients with advanced CRCs.

In this study, we evaluate the efficacy and safety of GV1001 vaccination in combination with chemotherapy as a second-line treatment for patients with metastatic CRCs.

## Methods

### Study design and patient eligibility

This study was a multicenter, single arm, phase 2 trial done at 7 hospitals in South Korea.

Patients were eligible for this study if they fulfilled all of the following criteria: (1) pathologically confirmed recurrent, or metastatic colorectal cancer patients who failed fist-line chemotherapy (oxaliplatin or irinotecan containing regimen); (2) measurable disease, as defined using version 1.1 of the Response Evaluation Criteria In Solid Tumors (RECIST); (3) age ≥19 years; (4) Eastern Cooperative Oncology Group (ECOG) performance status of 0-2; (5) life expectancy ≥ 12 weeks; (6) adequate hematological, renal, and hepatic functions, as defined using an absolute neutrophil count of ≥1.5 × 10^9^/L, a platelet count of ≥100 × 10^9^/L, serum creatinine levels of ≤1.5 × upper limit of normal or creatinine clearance ≥50 mL/min, serum bilirubin ≤2 × UNL, aspartate aminotransferase and alanine aminotransferase levels of ≤2.5 ×; and (7) willingness to provide informed consent to participate in this study.

Patients were excluded based on the following criteria: (1) other previous or concurrent malignancies within the last 5 years, with the exception of cured basal cell carcinoma of the skin or carcinoma *in situ* of the uterine cervix; (2) presence of intracerebral metastases or meningeal carcinomatosis; (3) other clinically significant comorbid conditions, such as an active infection or severe cardiopulmonary dysfunction; (4) medication that might affect immunocompetence such as long-term steroids or other immunosuppressants for an unrelated condition.

### Treatment

The vaccine GV1001 consists of a synthetic peptide corresponding to the 16 amino acid residue 611 to 626 (EARPALLTSRLRFIPK) of human telomerase reverse transcriptase (hTERT) and is capable of binding to molecules encoded by multiple alleles of all three loci of HLA class II. GV1001 was manufactured by Samsung Pharmacy (Hwasung-si, Korea) and supplied by GemVax & KAEL (Seongnam-si, Korea).

The selection of second-line chemotherapeutic agents and targeted agents (bevacizumab, cetuximab or aflibercept) depended on the investigator's choice. 0.56 mg of GV1001 was injected intradermally on days 1, 3, 5 and 8 during the first cycle of chemotherapy, then once on day 1 of subsequent cycles. GV1001 was diluted with 0.3 ml of 0.9% normal saline and administered intradermally to the lower abdomen within 6 hours after dilution. This treatment was repeated every 2 weeks until treatment is discontinued due to the subject's request, toxicities, or disease progression.

### Serum eotaxin level test

To determine the relationship between eotaxin level and treatment response, we conducted eotaxin level test in patients who consented to the test. Peripheral blood was collected at the baseline, the first day of 2^nd^, 4^th^, 7^th^, 10^th^ cycles of treatment and analyzed using Bio-Plex^®^ 200 systems at the Seoul Clinical Laboratories.

### Delayed-type hypersensitivity test

Delayed-type hypersensitivity (DTH) test was performed to determine whether an immune response has been induced. The test was performed at the baseline and on the first day of 2^nd^, 4^th^, 7^th^ and 10^th^ cycles of chemotherapy. We continued the test until the result was positive. 0.08 mL of the remaining solution (Solution A) after preparation for GV1001 injection is diluted in 0.22 mL of normal saline with a concentration of about 0.7 mg/ml (Solution B). 0.15ml of solution B is extracted and administered intradermally on the opposite lower abdomen within 6 hours after GV1001 injection. If the erythema or induration is more than 5 mm, it is evaluated as positive.

### T-cell proliferation test

Peripheral blood mononuclear cells (PBMCs) were isolated from blood samples before the start of vaccination and on the first day of 2^nd^, 4^th^, 7^th^, and 10^th^ cycles of chemotherapy to conduct T-cell proliferation test. T-cell proliferation was detected by flow cytometry using carboxy fluorescein diacetate succinimidyl ester (CFSE) (eBioscience 65-0850). After thawing PBMCs in at the end of the cycle, 1~5 × 10^6^ cells were incubated with 2mM CFSE at RT for 10min, washed with ice-cold completed RPMI1640 medium. 1 × 10^5^ CFSE stained cells were seeded in an anti-Human CD3 (1 mcg/ml) coated 96well culture plate in completed RPMI1640 medium. CFSE labeled PBMCs were stimulated with anti-Human CD28 (1 mcg/mL), GV1001 peptide (20 mcg/mL) in the anti-Human CD3 coated 96 well culture plate. PBMCs were incubated at 37 °C, 5% CO2 for 72h. Dividing cells were detected by flow cytometry and analyzed using CYTOFLEX software (Beckman).

A positive proliferative T-cell response was defined if one of the following criteria was met: i) a stimulation index (SI) ≥ 2 (SI was calculated by dividing T-cell population after GV1001 injection by that of the baseline value); ii) the difference in the number of T-cell division before and after GV1001 injection ≥ 1.

### Endpoints

The primary endpoint was disease control rate (DCR) and secondary endpoints were overall response rate (ORR), progression-free survival (PFS), OS and toxicity profiles. DTH test and T-cell proliferation test was performed to evaluate immune response which was the exploratory endpoint.

### Statistical analysis

According to Simon's optimal two-stage design, 46 patients were required for enrollment to test the null hypothesis that the true DCR is 30% versus the alternative hypothesis that the true DCR is at least 50%, with two-sided alpha of 0.10 and 90% power. If 7 patients or more with disease control (complete response + partial response + stable disease) were observed among 22 patients in the first stage, the study was continued with 24 additional patients included. As the drop-out rate was assumed to be 20%, the number of patients necessary for recruitment into the study was calculated to be 57.

Descriptive statistics were used to summarize the patients' characteristics, tumor responses, and safety events. The Kaplan-Meier method was used to estimate the median PFS and OS. All patients who received at least one cycle of treatment were included in the safety analysis and those who underwent at least one response evaluation were defined as modified intent-to-treat (mITT) population and included in the efficacy analysis.

## Results

### Patient characteristics

From May 13, 2015 to October 13, 2020, 56 patients with recurrent or metastatic CRC treated in seven hospitals of South Korea were enrolled. Table 1 shows the baseline characteristics of all patients. The median age was 64 years (range, 29-82 years) and 67.9% were men. Of all patients, 92.8% exhibited ECOG performance status 0-1. The primary tumors were predominantly located in the left side of the colon (left- vs. right-sided, 66.1% vs. 33.9%) and the RAS mutation was present in 25% of patients.

### Efficacy outcomes

**Figure 1:**
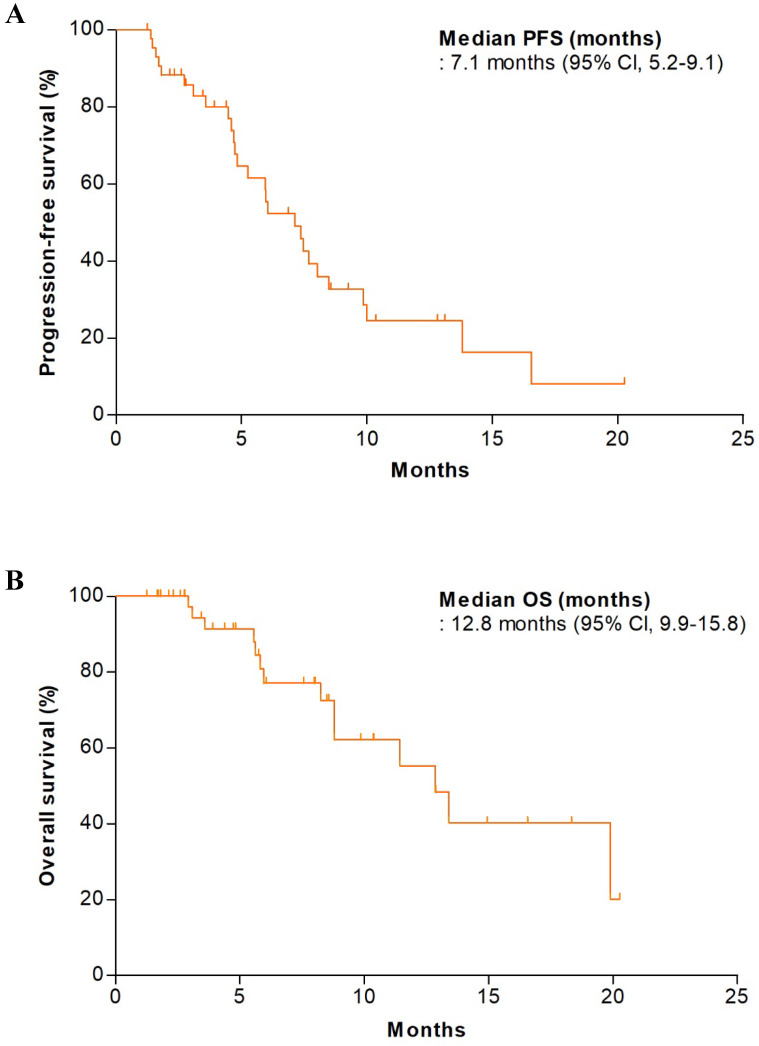
Kaplan-Meier estimates of **(A)** progression-free survival and **(B)** overall survival (mITT population, n=44).

In the first stage of the study, the DCR in the initial 22 patients was 81.8% (18 of 22); we thus proceeded to the second stage. Of all patients, efficacy analysis was performed on the mITT population; 12 patients were excluded because they received no treatment or did not undergo any tumor assessment. In the mITT population (n = 44), the DCR and ORR were 90.9% (95% CI, 82.4-99.4) and 34.1% (95% CI, 20.1-48.1), respectively (Table 2). One patient exhibited a complete response (2.3%). The median PFS was 7.1 months (95% CI, 5.2-9.1 months) and the median OS was 12.8 months (95% CI, 9.9-15.8 months) (Figure 1). We performed efficacy analysis according to the baseline eotaxin levels of all available patients (n = 22); patients with lower eotaxin levels (< 80 pg/mL) showed trends toward better efficacy outcomes (compared to patients with higher eotaxin levels [≥ 80 pg/mL]) in terms of the DCR (100.0% vs. 85.7%, p = 0.481) and the ORR (64.3% vs. 28.6%, p = 0.128) (Table 3), but there was no statistical significance.

### Safety outcomes

Table 4 lists the treatment-related adverse events. All-grade neutropenia (48.2%), anemia (42.9%), nausea (26.8%), neuropathy (25.0%), stomatitis (21.4%), and diarrhea (21.5%) were common (n = 56). The most common grade ≥ 3 adverse event was neutropenia (16.1%).

### Prognostic factors

We performed univariate and multivariate analyses to identify factors potentially prognostic of PFS and OS (Table S1). The multivariate analysis included factors with p-values < 0.5 in the univariate analyses. In the mITT population, two factors were independently associated with a poor PFS in multivariable analysis: age ≥ 65 years (hazard ratio for PFS, 3.37 [95% CI, 1.34-8.49], p = 0.010) and ECOG performance status 1 or 2 (hazard ratio for PFS, 2.6 [95% CI, 1.01-6.69], p = 0.048).

### DTH and T-cell proliferation tests

**Figure 2:**
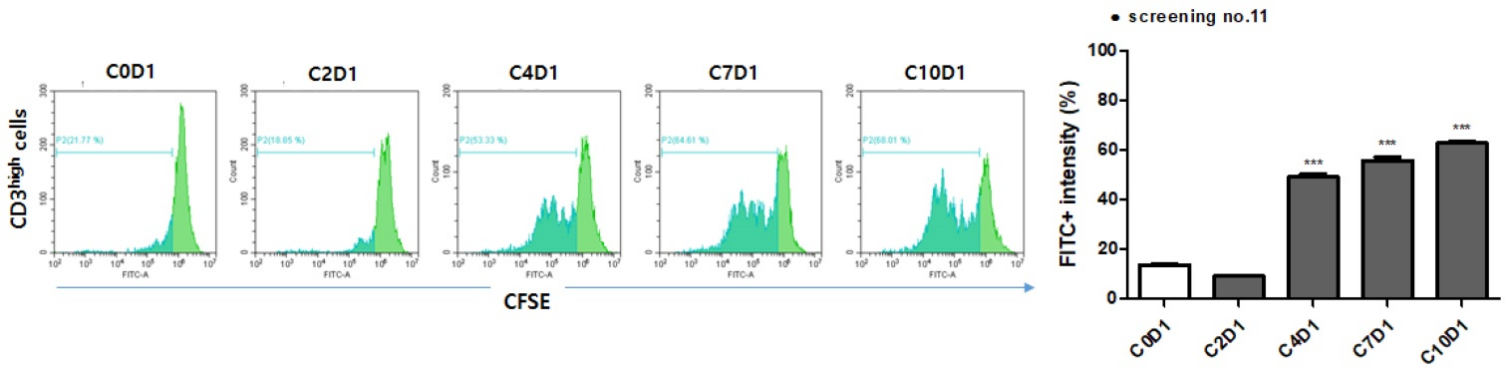
*In vitro* T cell proliferation in PBMC before the vaccination (C0D1) and after GV1001 vaccination (cycles 2, 4, 7 and 10). Histogram plots showing the division peaks following anti-CD3 (1 µg/mL), anti-CD28 (1 µg/mL), and GV1001 (20 µg/mL) stimulation of carboxy fluorescein diacetate succinimidyl ester (CFSE)-labeled CD3^high^ cells. Significance was evaluated by one-way ANOVA. **p < 0.01, and ***p < 0.001. In patient no. 11, T cells started to divide (histogram) from the fourth cycle of the GV1001 vaccination, and the CFSE intensity of CD3^high^ cells also significantly increased from the fourth cycle to the tenth cycle of treatment.

DTH results were available for 20 patients; no patients exhibited positive results during treatment. T-cell proliferation tests were conducted on 25 patients; GV1001-specific T-cell proliferation was evident in 7 (28.0%). The positive result of one patient is shown in Figure 2. Neither the ORR (42.8% in the positive vs. 53.3% in the negative group, p = 0.943) nor the DCR (100.0% in the positive vs. 88.9% in the negative group, p = 1.000) differed between the T-cell proliferation-positive and -negative groups. The median PFS (8.5 months [95% CI, 3.0-13.9 months] vs. 4.7 months [95% CI, 2.5-6.9 months], p = 0.303) tended to be longer in the T-cell proliferation-positive group, but this difference was not statistically significant. The median OS could not be analyzed in this subgroup because of the censored data.

## Discussion

In this study, we evaluated the efficacy and safety of GV1001 combined with chemotherapy in CRCs patients. To our knowledge, this is the first study to test a telomerase vaccine in patients with CRC.

In the results, the DCR was 90.9% (95% CI, 82.4-99.4); this was higher than the predefined value for proof of efficacy. The median PFS and OS (secondary endpoints) were 7.1 months (95% CI, 5.2- 9.1 months) and 12.8 months (95% CI, 9.9-15.8 months); these were comparable to the values in pivotal studies of second-line chemotherapies for CRCs 21, 22. However, no obvious immune response (on the DTH or T-cell proliferation test) was observed, in contrast to other GV1001 trials with pancreatic and non-small cell lung cancers. No patient exhibited positive results on the DTH test; antigen-specific T-cell proliferation was observed in only 28% of patients (7 of 25). In addition, the results of the T-cell proliferation test were not correlated with the efficacy outcomes. A discrepancy between the DTH reactions and T-cell responses was also observed in a previous study on pancreatic cancer patients 12 and a plausible explanation is suggested that different sensitivities in the two assays or biologically different immune reactions generated by vaccination might have influenced the outcomes. Considering our results, it is difficult to clearly determine whether the observed efficacy is attributable to the synergistic effect of GV1001 vaccination and chemotherapy or to chemotherapy itself.

Unlike other studies of GV1001, we did not combine injections of granulocyte macrophage colony-stimulating factor (GM-CSF) with GV1001 vaccination, which may explain why we did not observe an obvious immune response. In general, a level of immunogenicity that breaks the immune tolerance of the host is essential for a cancer vaccine to be effective; concomitant delivery of adjuvant GM-CSF with a vaccine is widely adopted strategy. Although GM-CSF-based vaccines induced potent anti-tumor immune responses in preclinical studies 23, 24, the effects were not robust in clinical trials; they sometimes contradicted the results from animal models 25, 26. Under certain conditions, GM-CSF induces the production of myeloid-derived suppressor cells and immunosuppressive regulatory T cells, leading to unexpected outcomes. Based on the fact that adding GM-CSF to vaccination could induce immunosuppression 27, we hypothesized that GV1001 vaccination alone (i.e., without GM-CSF injection) might induce an adequate immune response. However, it is possible that the omission of GM-CSF may have compromised immunogenicity.

Moreover, when cytotoxic chemotherapy is used in conjunction with anti-cancer vaccination, the chemotherapy itself is immunosuppressive and can thus affect antigen-specific T-cell responses. Gemcitabine and fluorouracil, the combination partners of GV1001 in phase III trial of pancreatic cancer, have preclinical evidence for synergism with GV1001 that these agents induce apoptosis of cancer cells leading to the release of antigens which can be taken up by antigen-presenting cells and cross-presented to cytotioxic T cells 28, 29. However, there is lack of evidence that oxaliplatin and irinotecan (the drugs used in the present study) act synergistically with GV1001.

Eotaxin is an eosinophil-specific chemokine associated with allergic reactions 30. In general, chemokines have important roles in cancer progression that involve modulating tumor cell growth and migration 31. However, little is known regarding the role of eotaxin in cancer. After the predictive value of eotaxin for GV1001 vaccination has been proposed in TeloVac study 15, consistent results were obtained in a subsequent phase III study of pancreatic cancer patients with high eotaxin levels 16. However, in this study, there was no significant relationship between baseline eotaxin level and efficacy outcomes. Because no obvious immune response was induced, it is difficult to clearly explain a possible role for eotaxin as a predictive marker in this study. In addition, considering the conflicting results of the role of eotaxin in various types of cancer, the role of eotaxin as a predictive marker for GV1001 in pancreatic cancer as well as in other cancers needs to be further verified. Indeed, eotaxin level was associated with a poor prognosis in certain types of cancer 32; conversely, it was associated with tumor suppression in other cancers 33.

## Conclusion

Although no obvious immune response was observed, this first clinical study of telomerase vaccination for CRC patients showed that GV1001 vaccination in combination with conventional chemotherapy was tolerable and associated with modest efficacy outcomes. More robust studies are required to validate a potential role for GV1001 in CRC treatment.

## Supplementary Material

Supplementary table.

## Figures and Tables

**Table 1:** Baseline characteristics of patients (All patients, n=56)

	Number of patients (%)
**Age, years**	
Median (range)	64 (29-82)
**Sex**	
Male	38 (67.9%)
Female	18 (32.1%)
**ECOG performance status**	
0	32 (57.1%)
1	20 (35.7%)
2	4 (7.1%)
**Disease status**	
Initially metastatic	39 (69.6%)
Recurrent	17 (30.4%)
**Site of primary tumor**	
Ascending colon	8 (14.3%)
Transverse colon	7 (12.5%)
Descending colon	5 (8.9%)
Recto-simoid colon	32 (57.1%)
NA	4 (7.1%)
**Histological differentiation**	
Well/moderate	46 (82.1%)
Poor/undifferentiated	8 (14.3%)
NA	2 (3.6%)
**Previous first-line treatment**	
FOLFOX/FOLFIRI + cetuximab	15 (26.8%)
FOLFOX/FOLFIRI + bevacizumab	21 (37.5%)
FOLFOX/FOLFIRI	20 (35.7%)
**RAS mutation**	
Wild type	32 (57.1%)
Mutant	14 (25.0%)
NA	10 (17.9%)
**Number of metastatic site**	
1-2	34 (60.7%)
≥3	10 (17.9%)
NA	12 (21.4%)

ECOG: Eastern Cooperative Oncology Group; NA: not available; FOLFOX: 5-fluorouracil, leucovorin, and oxaliplatin; FOLFIRI: 5-fluorouracil, leucovorin, and irinotecan.

**Table 2:** Best overall response (mITT population, n=44)

	mITT population (n=44): Number of patients (%)
**Best overall response**	
Complete response (CR)	1 (2.3%)
Partial response (PR)	14 (31.8%)
Stable disease (SD)	25 (56.8%)
Progressive disease (PD)	4 (9.1%)
Objective response rate^a^	34.1% (95% CI, 20.1-48.1)
Disease control rate^b^	90.9% (95% CI, 82.4-99.4)

^a^ Objective response rate is defined as the proportion of patients with CR or PR as best overall response; ^b^ Disease control rate is CR+PR+SD (including non-CR/non-PD). mITT: modified intention to treat; CI: confidence interval.

**Table 3:** Best overall response by baseline eotaxin level (n=28)

	Eotaxin high (≥80 pg/ml) (n=14)	Eotaxin low (<80 pg/ml) (n=14)	P value
**Best overall response**			
Complete response (CR)	0 (0.0%)	1 (7.2%)	
Partial response (PR)	4 (28.6%)	8 (57.1%)	
Stable disease (SD)	8 (57.1%)	5 (35.7%)	
Progressive disease (PD)	2 (14.3%)	0 (0.0%)	
Objective response rate^a^	28.6% (95% CI, 4.9-52.3)	64.3.0% (95% CI, 39.2-89.4)	0.128
Disease control rate^b^	85.7% (95% CI, 67.4-100.0)	100.0% (95% CI, 100.0-100.0)	0.481

^a^ Objective response rate is defined as the proportion of patients with CR or PR as best overall response;^b^ Disease control rate is CR+PR+SD (including non-CR/non-PD). CI: confidence interval.

**Table 4:** Treatment-related adverse events (all patients, n=56)

Treatment-related adverse events	All patients (n=56): Number of patients (%)	
	All grade	≥Grade 3
**Hematologic adverse events**		
Leukopenia	18 (32.1%)	1 (1.8%)
Neutropenia	27 (48.2%)	9 (16.1%)
Febrile neutropenia	0 (0.0%)	0 (0.0%)
Anemia	24 (42.9%)	0 (0.0%)
Thrombocytopenia	10 (17.9%)	0 (0.0%)
**Non-hematologic adverse events**		
Anorexia	10 (17.9%)	0 (0.0%)
Nausea	15 (26.8%)	4 (7.1%)
Vomiting	8 (14.3%)	4 (7.1%)
Stomatitis	12 (21.4%)	0 (0.0%)
Neuropathy	14 (25.0%)	2 (3.6%)
Diarrhea	12 (21.4%)	1 (1.8%)
Infection	3 (5.4%)	1 (1.8%)
Skin rash	5 (8.9%)	0 (0.0%)
AST/ALT increased	3 (5.4%)	0 (0.0%)
