# Supplementary material for: A phase II study of chemotherapy in combination with telomerase peptide vaccine (GV1001) as second-line treatment in patients with metastatic colorectal cancer
Source: J Cancer. 2022 Feb 14;13(4):1363–9. doi: 10.7150/jca.70385 (PMC8899359; doi:10.7150/jca.70385)
Supplement: Supplementary file 1 — Supplementary table. [file jcav13p1363s1.pdf]

Table S1. Univariable and multivariable analyses for progression-free survival and overall survival in mITT patients (n = 44)

	Progression-free survival				Overall survival			
	Univariable		Multivariable		Univariable		Multivariable	
	HR (95% CI)	P-value	HR (95% CI)	P-value	HR (95% CI)	P-value	HR (95% CI)	P-value
Age (years)								
<65	ref		ref		ref		-	-
≥65	2.32 (1.04-5.14)	0.039	3.37 (1.34-8.49)	0.010	1.34 (0.34-5.30)	0.673	-	-
Sex								
Male	ref		Ref		ref		-	-
Female	1.40 (0.65-3.03)	0.386	1.23 (0.54-2.81)	0.620	1.10 (0.36-3.32)	0.870	-	-
ECOG performance status								
0	ref		Ref		ref		ref	
1 or 2	1.88 (0.84-4.17)	0.124	2.60 (1.01-6.69)	0.048	4.24 (1.18-15.21)	0.027	3.77 (0.80-17.89)	0.094
Disease status								
Recurrent	Ref		Ref		ref		ref	
Initially metastatic	1.54 (0.67-3.55)	0.306	1.38 (0.58-3.31)	0.466	2.13 (0.59-7.68)	0.246	1.03 (0.26-4.10)	0.962
Site of primary tumor								
Other	ref		-	-	ref		-	-
Rt-sided	1.12 (0.37-3.36)	0.838	-	-	1.40 (0.37-5.29)	0.622	-	-

Histological differentiation								
Poor/undefferentiated	ref		-	-	ref		-	-
Well/moderate	1.35 (0.39-4.63)	0.635	-	-	0.78 (0.17-3.56)	0.752	-	-
Previous first-line treatment								
Others	ref		-	-	ref		-	-
FOLFOX/FOLFIRI + Cetuximab	1.22 (0.53-2.82)	0.639	-	-	1.36 (0.45-4.11)	0.587	-	-
RAS mutation								
Wild type	ref		-	-	ref		-	-
Mutant	1.38 (0.52-3.66)	0.518	-	-	0.55 (0.06-4.64)	0.581	-	-
Number of metastatic site								
1-2	ref		-	-	ref		ref	
≥3	1.46 (0.40-5.23)	0.566	-	-	2.18 (0.47-10.24)	0.322	4.74 (0.70-32.09)	0.111
